# Lineage Reconstruction of In Vitro Identified Antigen-Specific Autoreactive B Cells from Adaptive Immune Receptor Repertoires

**DOI:** 10.3390/ijms24010225

**Published:** 2022-12-23

**Authors:** Peter Blazso, Krisztian Csomos, Christopher M. Tipton, Boglarka Ujhazi, Jolan E. Walter

**Affiliations:** 1 Department of Pediatrics, University of Szeged, 6720 Szeged, Hungary; 2 Division of Pediatric Allergy/Immunology, University of South Florida at Johns Hopkins All Children’s Hospital, St. Petersburg, FL 33701, USA; 3 Department of Medicine, Division of Rheumatology, Emory University, Atlanta, GA 30322, USA; 4 Division of Allergy and Immunology, Massachusetts General Hospital for Children, Boston, MA 02114, USA

**Keywords:** B cell lineage, autoreactive B cells, autoimmunity, AIRR, IgH repertoire, monoclonal antibody, RAG deficiency, SLE

## Abstract

The emergence, survival, growth and maintenance of autoreactive (AR) B-cell clones, the hallmark of humoral autoimmunity, leave their footprints in B-cell receptor repertoires. Collecting IgH sequences related to polyreactive (PR) ones from adaptive immune receptor repertoire (AIRR) datasets make the reconstruction and analysis of PR/AR B-cell lineages possible. We developed a computational approach, named ImmChainTracer, to extract members and to visualize clonal relationships of such B-cell lineages. Our approach was successfully applied on the IgH repertoires of patients suffering from monogenic hypomorphic RAG1 and 2 deficiency (pRD) or polygenic systemic lupus erythematosus (SLE) autoimmune diseases to identify relatives of AR IgH sequences and to track their fate in AIRRs. Signs of clonal expansion, affinity maturation and class-switching events in PR/AR and non-PR/AR B-cell lineages were revealed. An extension of our method towards B-cell expansion caused by any trigger (e.g., infection, vaccination or antibody development) may provide deeper insight into antigen specific B-lymphogenesis.

## 1. Introduction

### 1.1. Formation of B Cell Lineages Is a Constant Somatic Microevolution

Maintenance of functional humoral immunity can be seen as a constantly ongoing evolution of diverse, functional and highly effective collection of antigen receptor expressing B cells. They can recognize and bind to any potential antigens occurring in nature while self-antigens must be tolerated. In this regard, mechanisms that continuously provide genetic diversity as well as negative and positive selection must exist.

B-lymphogenesis starts with the production of a genetically vastly diverse pool of naïve B cells in the bone marrow. Their heterogeneity originates from the strictly controlled V(D)J recombination events of the variable (V) regions of immunoglobulin heavy (IgH) and light (IgL) chain genes [1]. At the beginning of B-lymphoid development, original, germline variants of IgH and IgL genes are inactive, and they need to be rearranged first from sets of V (variable), D (diversity) and J (joining) gene segments in case of IgH on chromosome 14. Only V and J segments on chromosome 2 (kappa chain) or 22 (lambda chain) are used for IgL assembly. One segment from each region is selected and cut randomly by the recombination-activating gene 1 and 2 (RAG1/RAG2) enzyme complex. After cut 5′ and 3′ termini of DNA fragments form closed hairpin structures, they are opened up usually asymmetrically by the endonuclease Artemis. This leaves the strands of DNA fragments with different lengths. Shorter strands are filled by adding complementary nucleotides (named P-nucleotides) opposite to the overhanging ones. Moreover, random number (up to 20) and type of extra nucleotides (named N-nucleotides) are added to the blunt(ed) sequences by the terminal deoxynucleotidyl transferase (TdT) at the junctions between V-J and J-D segments in IgH, and V-D sequences in IgL. Selected and modified segments are ligated by the non-homologous end-joining (NHEJ) DNA repair machinery for both IgH and IgL chain assembly. Two IgH-IgL subunit pairs are covalently attached together resulting in a highly unique, cell-specific B-cell receptor (BCR) protein complex. BCR is expressed on the surface of immature naïve B lymphocytes as either IgD or IgM type membrane-bound immunoglobulin. Mature, naïve B cells reach the periphery and when their BCR recognize an antigen they become activated. Activated B- cells receive help from CD4+ helper T cells and undergo rapid proliferation in the germinal centers (GC) of follicles, where somatic diversification (class-switch recombination, CSR; and somatic hypermutation (SHM)) take place and then they differentiate to either plasma cells or memory B cells. CSR affects the constant (C) region of the IgH gene, and it is responsible for the subsequent isotype change of IgM to IgG, IgA or IgE [2]. During the germinal center reaction, CSR is achieved by concerted enzymatic actions of activation-induced cytidine deaminase (AID), uracil N-glycosylase (UNG), apurinic/apyrimidinic endonuclease 1 (APE1) that leads to double strand DNA breaks in C region. Repair of these breaks by NHEJ results in the replacement of mu sequence in the C region with gamma, alpha or epsilon sequence for IgG, IgA or IgE isotypes, respectively. During SHM, B cells accumulate point mutations in the V regions of their IgH and IgL at a very high rate (1/1000 nucleotides per cell cycle) due to the actions of AID and subsequent different base excision or mismatch repair pathways [3]. Rarely—approximately one in every 10,000 nucleotides—insertions or deletions are also introduced [4,5]. Together, these events enable affinity maturation, an iterative positive selection process, which ends up in the generation of stronger binding BCRs and antibodies. Alternatively, B cell activation may occur outside of the follicles with or without of T cell help, a process known as extrafollicular (EF) B-cell differentiation.

Diversity of the antibodies come from the above detailed four sources: cutting and pasting of random V, D, J fragments (combinatorial diversity), random composition of extra DNA sequences inserted between these fragments (junctional diversity), class switch recombination (different isotypes) and nucleotide substitutions or insertions/deletions generated by SHM during affinity maturation (mutational diversity). The stochastic nature of these processes on one hand establishes the huge variety as well as increasing affinity of specific BCRs or antibodies but on the other hand continuously generates poly- or autoreactive (PR/AR) antibodies. Therefore, B cells must go through multiple rounds of negative and positive selection before they are allowed to maintain the humoral immune homeostasis of the body.

### 1.2. Humoral Autoimmunity Is Interpreted as Evolving Autoreactive B Cell Lineages

Negative selection—or also known as tolerance filters—guarantees that B lymphocytes and their descendants tolerate self-antigens.

In the bone marrow, the central generative organ of B cells, a wide array of autoantigens is presented to the developing early B cells. If BCR engagement by a self-antigen is weak, the immature B lymphocyte becomes unresponsive (anergic) to repeated antigen binding. If BCR binds to self-structures (especially on the surface of other cells) with high affinity, the AR BCR bearing B cell is either removed by apoptosis (deleted) or another round of IgL gene rearrangement is completed by re-activating V(D)J recombination. The latter is called receptor editing and aims to replace the light chain of self-recognizing BCR that thus may become less- or non-AR. As a consequence of these filtering steps—also named as central tolerance—efflux of functional AR mature naïve B cells into the circulation is significantly reduced though not completely prevented [6].

**Figure 1:**
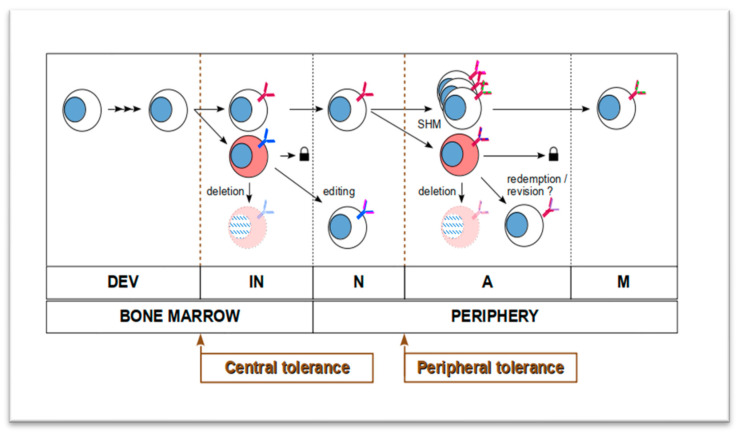
Mechanisms that shape the B-cell repertoire and B-cell lineages. Weakening of negative selective pressure (tolerance mechanisms) on B cells make the emergence and persistent survival of “intolerant” autoaggressive B cells possible. DEV: early developmental stage of lymphoid lineage from hematopoietic stem cells in the bone marrow; IN: immature naïve B cells undergoing central selection mechanisms (tolerance); N: naïve B cells that left bone marrow; A: activated B cells in the periphery; M: memory B cells; SHM: somatic hypermutation. Padlocks refer to inactivation or anergy.

If a mature naïve B cell encounters a self-structure with low affinity at the periphery, it will be either not respond or be inactivated (anergy). Anergic B cells can still be reactivated but they need elevated concentration of B-cell activating factor (BAFF) to do so. If BCR binds an autoantigen with higher affinity, its expressing B lymphocyte will die by apoptosis (deletion) or be inhibited via co-receptor signaling (FcγRIIB and CD22). Additionally, regulatory T-cells (Tregs) can directly suppress autoreactive B cells or limit the repertoire of follicular helper T-cell presented antigens to decrease the chance of AR B cell activation [7,8]. Several studies suggest that other mechanisms exist too, that help attenuate autoreactivity of the peripheral B lymphocyte pool. One mechanism is receptor revision, which refers to the re-expressing and re-applying V(D)J recombination machinery in a mature B cell to restrict autoreactivity by rearranging and using a new V region [9,10,11,12]. Another mechanism is clonal redemption, which utilizes SHM to mutate the V region of an autoantibody away from self-antigen binding and increases binding affinity to a foreign antigen [13,14]. In the end, these mechanisms occur outside of the bone marrow; hence, they enable peripheral tolerance, which ensures that no self-attacking B cells emerge and persist (see Figure 1).

Humoral autoimmunity appears when AR B cells escape from these tolerance filters, thrive and cause damage. Therefore, autoaggressive antibody responses are byproducts of the same, but erroneous evolutionary process of B lymphocytes that also generates foreign antigen specific antibodies [15]. Evolution can be interpreted as a history of adaptive changes in a lineage of organisms or cells. In lymphoid lineages, maturating and differentiating lymphocytes must always adapt to the restrictive environment set by central and peripheral tolerance. Depending on which component of this filtering system is impaired, it may affect different properties (survival, expansion, diversification) of B-cell lineages via leakage of self-targeting cells at various stages of their development [8,16,17].

### 1.3. Autoreactive B-Cell Lineages Leave Their Footprints in BCR Repertoires

Evolutionary histories of B-cell lineages are imprinted into the variable regions of heavy (IgHV) and light (IgLV) chain genes of their member cell clones. Th-independent/dependent activation, such as clonal expansion without/with diversification (SHM) [18] and isotype switch (CSR) [19] or clonal anergy/deletion all leave their marks on the composition of specific IgHV/IgLV sequences and/or count of carrier cells in any given lineage. The sum of these sequences in all or sorted group (compartment) of B cells is also referred to as the adaptive immune receptor repertoire (AIRR). Next-generation sequencing (NGS) technology has now made the reading and analysis of AIRRs possible via massively parallel sequencing of the IgH/IgL gene from usually 10^4^–10^6^ sorted B cells (AIRR-seq). This provides sufficient depth and detail for the investigation of most dominant B-cell lineages. Their members can be collected by clustering closely related IgH/IgL sequences in an AIRR dataset with acceptable reliability [15,20,21,22]. 

True AR B lymphocytes are not straightforward to identify within these datasets. Self-reactive IgH/IgL clones are not easily distinguishable from others based solely on their nucleotide sequences [23]. Scarce cases have been documented when a defined gene usage/nucleotide sequence intrinsically determines self-reactivity. For example, unmutated IgVH4-34 gene segment is known to recognize i/I self-antigen on the surface of the red blood cells by two motifs (Q^6^W^7^ and A^24^V^25^Y^26^) within its first framework region (FR1) and the expansion of B cells carrying these motifs is associated with autoimmune hemolytic anemia [24,25,26]. However, there is no accurate detection available in the majority of humoral autoimmunity cases. One of the reasons might be the uncoupled nature of IgH and IgK/L chains in bulk AIRR-seq data. IgH and IgK/L must be viewed together since the change of either one—such as during receptor editing/revision—is enough to weaken/block affinity of the antibody to autoantigen. Most recent single-cell sequencing approaches can overcome this limitation but the uncertainty in binding affinity and antigen specificity of hypothetically self-targeting Ig sequences still remains.

Representative marker of humoral autoreactivity has already been established in a transgenic Nur77-eGFP reporter mouse model [7,27] and led to the ability to track PR/AR B cells and confirm anergy in the mature naïve compartment. For ethical, legal and health concerns, such an approach in humans is not applicable. In our study we aimed the tracking of IgH sequences of in vitro characterized single-cell cloned PR antibodies in human AIRRs. For this, we reused data from already published datasets either derived from monogenic RAG1 and 2 deficient or polygenic SLE patients [16,18,28,29].

## 2. Results

### 2.1. PR/AR IgH-Related Sequences Were Extracted from AIRRs of pRD Patients

**Figure 2:**
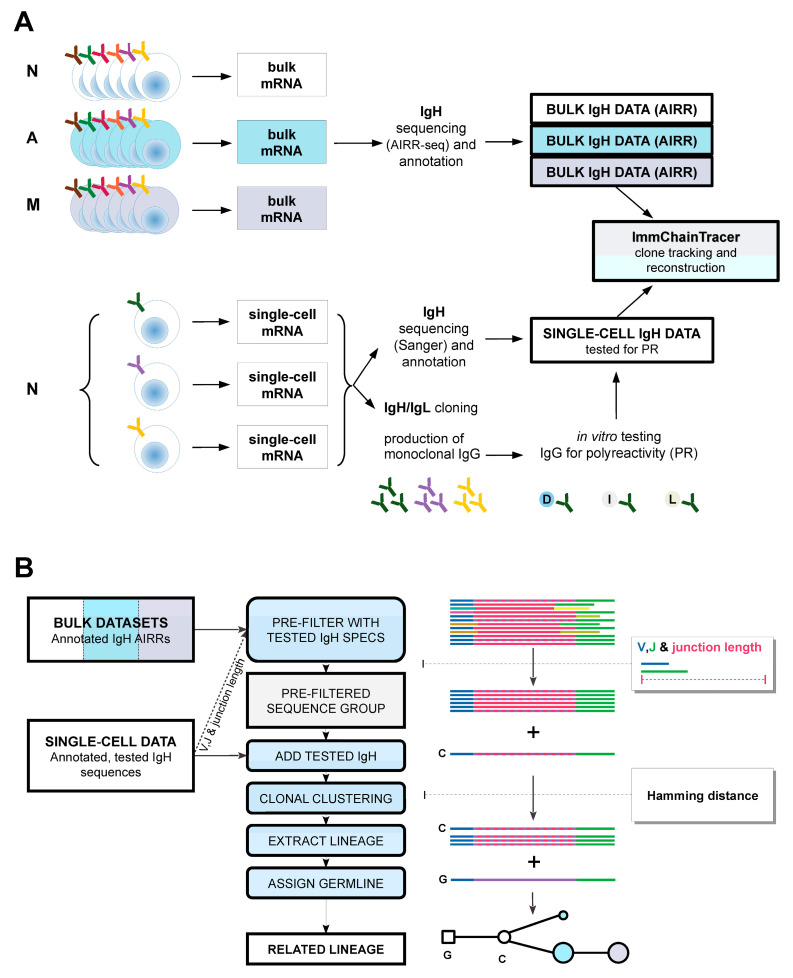
Schematic representation of sample preparation and processing. (**A**) Panel depicts wet-lab sample preparation and sequencing strategies combined with bioinformatic data processing. Initially, B cells are separated by a cell sorter based on their immunophenotypes (N—mature naïve; A—atypical naïve; M—memory). Prior to targeted RNA sequencing (AIRRseq) bulk messenger RNA is purified from each, sorted compartment. From the naïve compartment of the same subject single B-cell derived monoclonal antibodies are cloned, their heavy/light chains are sequenced, re-expressed and are tested for auto-/polyreactivity. This latter procedure is based on the original method published by Wardemann et al. [31]; (**B**) ImmChainTracer (ICT) pipeline. Previously annotated and prepared IgH adaptive immune receptor repertoire (AIRR) datasets from preliminarily sorted B-cell compartments are first pre-filtered to match V-, J- gene annotations and size of a single naïve B-cell cloned IgH. This IgH sequence is added to the filtered repertoire. Pre-determined Hamming distance threshold is applied to perform an unsupervised clonal clustering on this repertoire. Clonotype group of the drop-in sequence is extracted. Putative unmutated common ancestor (UCA) or “germline” of the extracted clonotype is determined and added to this group which is eventually used for further analyses (e.g., lineage tree reconstruction).

In order to identify compartmental clonotype groups (relatives of a B lymphocyte in a specified compartment) of PR/AR B cells we applied the following approach. We developed and applied a novel computational method (ImmChainTracer or ICT) that uses single-cell derived IgH sequences of in vitro characterized—in our case polyreactive—antibodies to search for their relatives in bulk IgH repertoires of different compartments of the same subject (see Figure 2). ICT extracts IgH sequences from AIRR datasets that fall within a pre-determined genetic distance from an input IgH sequence and share the same V-, J-gene usage and junction length (see Figure 2B). In other words, ICT finds IgHs in a BCR heavy chain repertoire that are grouped together with the input sequence similarly to the already established clonotype clustering procedure in Change-O toolkit [30].

Altogether 99 (29 + 39 + 31) IgGs from three control healthy (C1-3) donors and 100 (33 + 27 + 40) IgGs from three pRD (P1-3) patients were used for further testing. Each of 199 recombinantly expressed antibodies was tested by ELISA to bind double stranded DNA, human insulin and lipopolysaccharide (LPS). Antibodies were labeled polyreactive/autoreactive (PR/AR) if they recognized at least two of these three autoantigens. All other antibodies not fulfilling this criterion were labeled non-polyreactive (NPR). 

**Figure 3:**
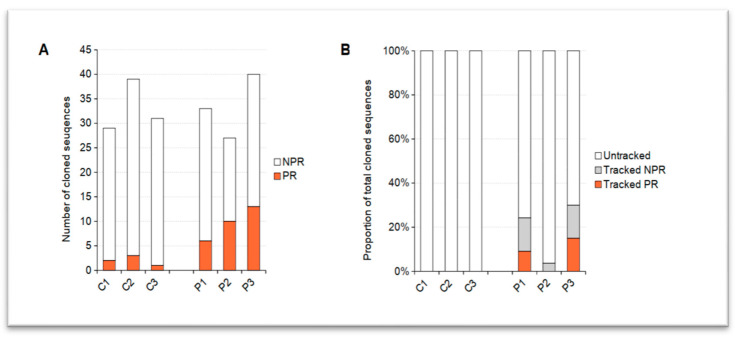
(**A**). Number and portion of polyreactive (PR/AR) and non-polyreactive sequences successfully cloned from single naïve B-cells of healthy control (C1–C3) or partially RAG1/2 deficient patients (P1-3); (**B**). Percentages of cloned IgH sequences (either PR/AR or NPR) that could be tracked in healthy control (C1–C3) or partially RAG1/2 deficient patients (P1-3).

PR/AR antibodies could be detected in both the pRD and healthy donor-derived samples (see Figure 3A). Presence of PR/AR antibody producing B cells with low frequency in the resting mature naïve compartment of healthy individuals is a known phenomenon [8,31]. PR/AR B cells has a wide range of specificity including the response against various external (e.g., common human pathogens) or internal (auto) antigens. Thus, it is also an important part of first-line defense mechanisms that makes the body clean of infections or dead cells [32]. Since significant portion of these PR/AR B cells/antibodies show AR properties they are continuously ablated by peripheral tolerance mechanisms. Therefore, expansion and increased longevity of this PR/AR B-cell pool can be an initial hallmark of humoral autoreactivity and weak(ened) tolerance processes. It has already been observed that pRD patients show a diverse set of autoantibody production and humoral autoreactivity [28]. As expected, we could detect higher number of PR/AR B-cell clonotypes (18%, 37% and 32%) in pRD patients compared to healthy donors (7%, 8% and 3%) (see Figure 3A). Finding increased proportion of mature naïve PR/AR B-cell clonotypes in such patients is consistent with the previously published data. 

### 2.2. Expansion of PR/AR-Related Clonotypes Were Detected in pRD Patients

Based on our previous studies [18,28] and current measurements, we postulated that due to the increase in supposed AR B-cell leakage through tolerance filters we would also find descendants of tested naïve PR/AR B cells in effector compartments as well. We managed to identify relatives of 24%, 3.7% and 30% of all tested (PR/AR and NPR) clonotypes in later-stage, non-naïve compartments of P1, P2 and P3 patients, respectively (see Figure 3B). Descendants of 9% and 15% of PR/AR B-clonotypes could be detected only in P1 and P3 subjects. Surprisingly, NPR-related IgHs were also found in all pRDs. Conversely, no relatives of any tested IgHs could be detected in healthy donors where the peripheral B-cell pools were made clean of PR/AR lineages. Together these results might also suggest the co-existence of some other factors (e.g., altered cytokine milieu that promotes B lymphocyte activation) in pRD subjects besides impaired tolerance mechanisms. This process may not allow for the emergence and persistence of PR/AR clonotypes only but it may also help other NPR B-cell lineages proliferate and thrive as it is seen here and suggested previously due to BAFF overproduction [29,33].

**Figure 4:**
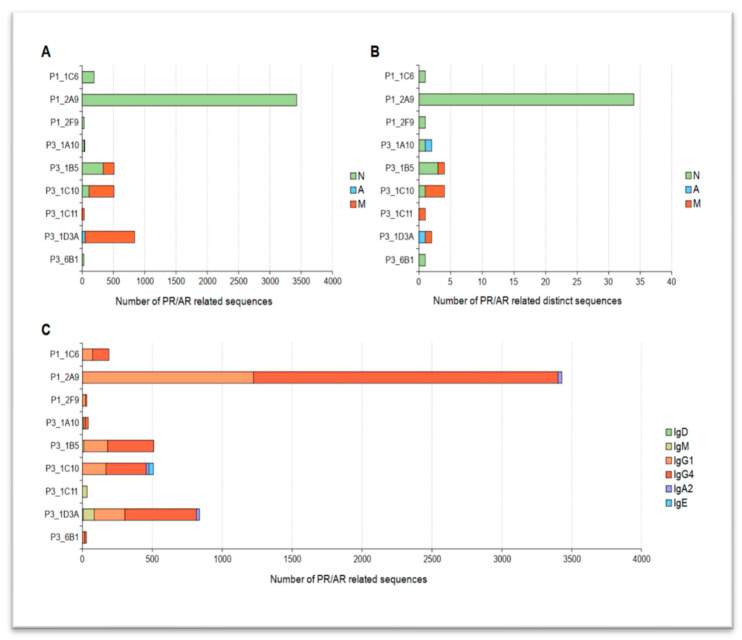
Expansion, diversification and isotype usage of PR/AR lineages. (**A**) Bars represent the total count of PR/AR related sequences in different compartments, (**B**) Bars represent the number of PR/AR related distinct sequences in different compartments of B cells (N: mature naïve, A: atypical naïve, M: memory); (**C**) Number of PR/AR related sequences sharing the same isotype. Isotypes are color-coded (see legend).

Close relatives of PR/AR IgHs show expansion in all investigated B-cell compartments (see Figure 4A). Interestingly, in both pRD patients (P1, P3), where related sequences were trackable, PR/AR descendants predominantly and widely spread out in the mature naïve compartment (100% and 66.7% of lineages affected, respectively). In only P3, relatives of 83.3% (5 out of 6) of PR/AR lineages contributed to the effector B-cell pools and four of them (66.7%) even reached the memory compartment as well. The fact that clonotype groups of IgH sequences with AR properties could be identified in the effector compartments implies the unrestrained production and longevity of autoantibody producing plasma cells.

### 2.3. Diversification and Class-Switching in PR/AR Lineages Imply Early Activation

As naïve B cells recognize their specific (auto) antigen, they become activated. It results not only in proliferation but also in affinity maturation of the IgH gene’s V region and CSR of the C region as detailed above. We questioned whether we could demonstrate such consequences of Th-cell dependent activation in the extracted PR/AR related clonotype groups.

To assess SHM activity, the number of distinct V regions of IgHs were counted (see Figure 4B) in each clonotype group. In P1, one in three mature naïve clonotype groups consists of 34 different V region sequences. In P3, where PR/AR B-cell progenies even reached effector compartments, two out of ten compartmental clonotype groups (one mature naïve and one memory) were made up of three distinct V sequences. Unexpectedly, in pRD patients, we saw signs of clonotype diversification early in the mature naïve subset. It might suggest that though these B cells were characterized as resting naïve (IgD^+^ CD21^+^ CD24^int^ CD27^−^ CD38^int^), a part of them may represent activated, antigen-experienced B cells and cannot be defined as real naïve cells. Isotype switch to IgGs or further could also be observed in eight out of nine lineages (Figure 4C). Moreover, in a single lineage IgE and in three groups IgA isotypes were also found. Altogether, both signs of SHM and class-switching imply (auto) antigen-driven activation in these PR/AR B clones.

### 2.4. Phylogeny Reveals Paths of PR/AR Antibody Maturation in pRD Patients

Numerous efforts have been made to generate authentic phylogeny trees of B-cell lineages from a group of Ig sequences [34,35,36,37,38]. These approaches are used to visualize and help understand the affinity maturation process of antibodies targeted against specific antigens (e.g., pathogens or vaccines). These methods can also be utilized on PR/AR-related sequences to demonstrate the history of the most significant changes a V-region of the antibody undergoes until the carrier B lineage reaches its final effector compartment. 

**Figure 5:**
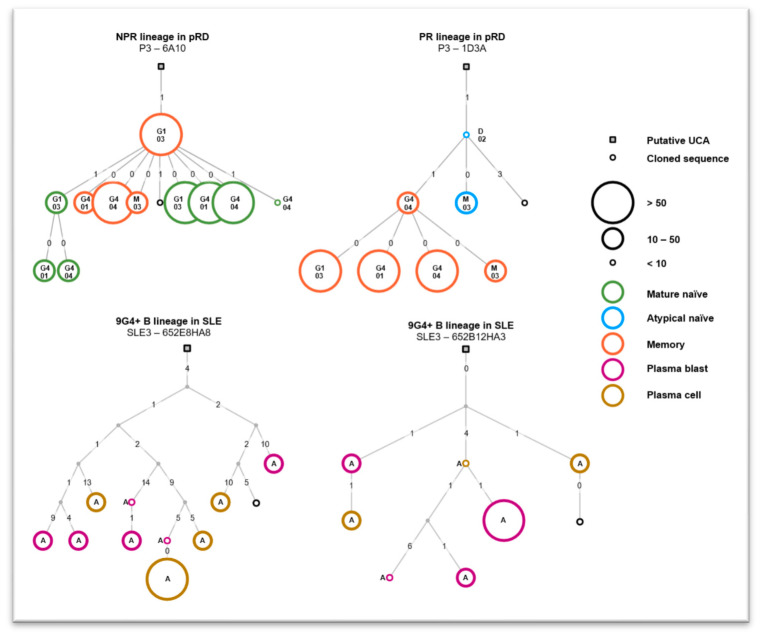
Representative phylogenetic trees of tracked B-cell lineages. Phylogeny of B-cell clonotypes are visualized as tree shaped graphs where nodes (rectangles or circles) represent distinct IgH sequences and edges (lines) show connections between two nodes. Node size refers to the number of identical IgH sequences. Node color marks compartmental affiliation (see legend). Labels within or next to nodes indicate isotype of nodal IgH. Numbers on connecting edges show the genetic Hamming distance between V regions of connected IgHs.

To the best of our knowledge, benchmarking of these algorithms has not been performed on PR/AR related clonotypes. Therefore, we applied the most frequently used maximum parsimony principle implemented in the Immcantation framework to reconstruct phylogenetic trees of our lineages [39,40]. Representative examples of a non-PR/AR and an PR/AR lineage from pRD patients show (see Figure 5) that though diversification took place during maturation, genetic distance is short (only 1–4 mutations) between the putative unmutated common ancestor (UCA) or the cloned naïve B-cell IgH and the final, potentially matured IgH descendant in the memory compartment. In other words, germline PR/AR B cells do not seem to mutate far away from the original properties throughout their lineage development and it suggests an extrafollicular fate; however, further investigation is needed. Additionally, early, predominantly centrifugal arborization of these lineages suddenly end in isotype switch, effector differentiation and expansion. The low number of tracked lineages make it difficult to draw firm conclusions from these observations, but possibly, the very early and stochastic (and not necessarily antigen-driven) activation of these B cells might explain these phenomena.

### 2.5. 9G4+ B Cells Also Expand and Thrive in SLE

Previously, it has been shown that already described 9G4 auto-/polyreactive antibody producing B cells in systemic lupus erythematosus proliferate and contribute to humoral autoreactivity [16,41]. In order to validate our approach and test its applicability in another, classical humoral autoimmune condition we re-analyzed single-cell and AIRRseq data from an SLE (identified as “SLE3.292”) patient suffering from high disease activity. 

Sixty-four single 9G4+ B-cell derived IgH sequences were tracked with ImmChainTracer on BCR IgH repertoires of six different peripheral B lymphocyte compartments of the flaring SLE patient. As assumed, we could detect closely related clonotype groups of single, 9G4+ B-cell clones. Nine related lineages could be tracked, reconstructed and analyzed by our algorithm (for two representative phylogeny trees see Figure 5). Seemingly, heavier mutation burden affects these trees compared to the ones in pRD patients. Additionally, more of the early ancestors are missing from these tracked lineages. Most of them are only present virtually as putative sequences probably because they have disappeared and been replaced by their mutated progenies. These observations shed light on the different characteristics (e.g., age or shaping mechanisms) of these lineages.

In summary, we could recapitulate and confirm prior findings stating that 9G4+ B cells not only expand and thrive but also class-switch and show signs of diversification. Moreover, these results help pave the way for broader utilization of our method.

## 3. Discussion

As shown here, clustering IgHs from bulk AIRRseq data that fall within close genetic proximity to in vitro characterized, single-cell derived poly/autoreactive heavy chain Ig sequences do aid the reconstruction of meaningful PR/AR lineages.

Deciphering molecular mechanisms of humoral autoimmunity is still a thoroughly investigated field in human immunology and rheumatology. If it is viewed from the evolutionary aspect of autoreactive lymphoid clonotypes, tools that aid the reliable tracking, reconstruction and characterization of such lineages seem indispensable for such research [42,43]. Polyreactive/autoreactive B cells circulating in peripheral blood do not necessarily initiate or contribute to autoimmune inflammation and self-damage. These cells escape tolerance filters, encounter their cognate auto antigen, become activated and more importantly, persist. Thus, exploring their genealogy is one of the keys to learn how, when and where they emerge and become active. We developed an algorithm (ImmChainTracer) that combined with a wet lab strategy (Figure 3A) helps the identification and fate tracking of tested PR/AR B cells. It was not only validated in SLE, a well-known humoral autoimmunity, but it was also proven useful in an unconventional autoimmune condition, partial RAG1/2 deficiency. 

Adding concurrent IgK/L tracking would further enhance the unfolding of fine details, since a potential IgK-L change (by receptor revision, SHM or other mechanisms) could also alter the biological behavior of PR/AR clones. However, the biological effects of light chain changes on the expansion and diversification properties of sub-clones have to be observable on IgH trees as well. Coupling IgH and IgK/L lineages are problematic because bulk AIRRseq data does not provide cellular affiliation of such sequences. Furthermore, faithful lineage reconstruction heavily depends on the precision of clonotype clustering. Up to now, different methods have been worked out to increase specificity and efficiency on bulk AIRRseq data [15,44,45], but none of them can guarantee that two identical or highly similar IgH or IgK/L sequences come from the same cell or lineage.

Single B-cell RNA sequencing (or sc-AIRRseq) is able to help overcome these obstacles since the origin of heavy (IgH) and light (IgK/L) immunoglobulin chains are readily defined by using cell-specific molecular barcode sequences [46,47]. Clonotype assignments and lineage tree reconstruction can be improved further by matching heavy with light chain trees and handling the outliers [48]. A single-cell approach also brings several other advantages over bulk sequencing. Coupled heavy and light chain genes from putative autoreactive lineages can be synthesized, re-expressed and antibodies from any cell can be verified in vitro. Single-cell RNAseq allows transcriptional profiling and examination of gene regulatory network changes within autoreactive clonotypes. Extent of clonal expansions is determined with higher precision since cell counts are based on unique cellular barcodes and not only on IgH expression levels in cell populations. Furthermore, integration of phenotypic profiles of single cells into the transcriptome is possible by staining the cells with oligonucleotide-labeled marker antibodies before sequencing [49]. Therefore, use of phenotypic marker antibodies will not be limited by the availability of dyes and properties of the flow cytometry instrument. This method, also called as CITE-seq, thus elevates the detail of characterization of such cells and lineages to an unprecedentedly high level.

Successful attempts of PR/AR lineage tracking in pRD and SLE encourage the extension of the presented method towards other—not just humoral autoimmune—diseases where emerging adaptive (auto) reactivity is or could be part or cause of the disease mechanism. Additionally, a more generalized utilization can be imagined in the fields of infectious diseases, vaccinology or biological therapy development as well. Observing, dissecting and/or optimizing pathways of single B-cell responses to specific antigens may contribute to manufacturing more effective vaccines or drugs in the future.

## 4. Materials and Methods

### 4.1. Preparation of Input IgH Sequences

Already published in vitro tested single-cell IgH sequences and bulk IgH repertoires from different peripheral B-cell compartments of pRD, SLE patients and healthy donors were used for further bioinformatic analyses. For more information on pRD patients (genetic and clinical background), see Supplementary Table S1. Wet lab processing of peripheral blood samples prepared for high-throughput, targeted RNA sequencing are detailed in these papers [16,18].

Briefly, peripheral blood mononuclear cells (PBMCs) of human subjects were isolated, followed by enrichment for B cells using CD20 positive selection (Milteny Biotec, Gaithersburg, MD, USA). B cells then were stained with viability dye to exclude dead cells, then a panel of B-cell markers (IgD, CD10, CD19, CD21, CD24, CD27 and CD38). Mature naïve B cells were identified as CD19^+^ IgD^+^ CD27^−^ CD38^int^ and either single sorted into 96-well PCR plates for cloning their expressed immunoglobulins or bulk-sorted for IgH repertoire sequencing. Total memory and atypical naive B cells were identified as CD19^+^ CD27^−^ and CD19^+^ IgD^+^ CD27^−^ CD38^low^ cells and bulk-sorted for repertoire sequencing. Similarly, naïve (CD19^+^IgD^+^CD27^−^), IgD^−^ memory cells (CD19^+^IgD^−^CD27^+^, including isotype-switched and immunoglobulin M (IgM)-only cells) and antibody secreting cells (ASCs, CD19^+^IgD^−^CD27^hi^CD38^hi^) were sorted from the peripheral blood of SLE patients for IgH repertoire sequencing. At least 5 × 10^4^ cells were collected from each population with a FACSAria II (BD Biosciences, Franklin Lakes, NJ, USA).

Bulk messenger RNA from such distinct B-cell pools were purified and used for targeted RNAseq (AIRRseq) to generate IgH repertoires representing each compartment. AIRR sequencing of pRD RNA samples was performed by iRepertoire (Huntsville, AL, USA). IgH repertoires of CD27^+^/^−^ B cells of the SLE patient were omitted from our subsequent analysis. Single-cell cloning, expression of coupled IgH and IgK/L sequences and testing recombinant antibodies for polyreactivity were performed as described before [18,31]. Both single-cell and bulk IgH sequences were re-annotated using IMGT/HighV-QUEST [50]. Subsequent data processing was performed with the Immcantation framework [30,40], Tidyverse system (H. Wickham and G. Grolemund, R for Data Science) [51] and the R language. IMGT annotated data were first converted to adaptive immune receptor repertoire (AIRR) rearrangement format [52] using “MakeDb.py” script of Change-O package. Isotypes and sequence counts were added from the initial iRepertoire data. For further analysis, FWR1 region was trimmed and only the CDR1-FWR4 region was kept. Distinct groups of identical sequences were merged into unique records summing their sequence counts. In order to alleviate the possible bias due to reverse transcription, amplification and sequencing errors, the following (“collapsing”) strategy was applied. Sequences with fewer than five copies were separated into a low-copy number collection keeping the high-copy number portion as well. Hamming distance was calculated between each low-copy and high-copy count sequences. If this distance between a low- and a high-copy pair was not larger than one, copy count of the low-copy sequence was added to the count of its high-copy pair. After this step the low-copy number pool of sequences was discarded. Finally, sequences labeled non-productive were filtered out of the retained, final high-copy pool. Total, productive, collapsed sequence counts and Hamming distance thresholds used for clonotype clustering of each repertoire are listed in Supplementary Table S2. IgH sequences of single-cell cloned, tested monoclonal antibodies were annotated by IMGT the same way as bulk sequencing data.

### 4.2. ImmChainTracer Pipeline

We developed a series of algorithms that clusters relatives of IMGT annotated, single-cell derived IgH sequences in specified AIRR datasets (see Figure 2B). It is part of our AIRRMINE data processing system that is also available online in an open source repository (https://github.com/blazsop/airrmine (accessed on 19 December 2022)). It also contains sample data and detailed documentation on its usage.

Preliminarily, Hamming distance threshold normalized by sequence length was calculated in the dataset for automated clonotype assignment For this, we initially used the ‘findThreshold’ method from SHazaM (v.1.0.2 package). Since it failed in a few cases of pRD patients, we further analyzed these cases to overcome this problem. This led to the development of our approach keeping the strategy of assessing a genetic distance threshold similar to the referred algorithm but eliminating the need for local minimum detection that failed in some of our pRD repertoires. Briefly, Hamming distance matrix of nearest neighbors normalized by lengths of IgH sequences was determined by the ‘distToNearest’ R function (mode, ‘ham’; normalize, ‘len’; and first, ‘FALSE’). The histogram of these distances was smoothed. The first non-declining value’s position after the first peak, instead of the local minimum (between two peaks), was assessed. Finally, this position as the distance threshold was used to generate clonotype groups utilizing the ‘DefineClones.py’ script (–mode gene–act set–norm len–model ham). Closest unmutated common ancestor (UCA) sequences were generated by the ‘CreateGermlines.py’ algorithm (-g dmask–cloned) and inserted into clonotype clusters.

Next, a pre-filtered repertoire was generated to decrease the size of the target repertoire for the time consuming clonotype clustering phase. For this, V-, J-gene annotations and junction length of the input single-cell DNA sequence were matched to exclude irrelevant sequences. Single-cell cloned, annotated IgH was added to this pre-filtered repertoire. In this combined data, clonotype assignment clustered related sequences around the input IgH. This way, the closest relatives of the input sequence were grouped together as a lineage. Prior to lineage tracking, we merged AIRR data of different compartments from the same subject to track B-cell lineages through developmental stages.

### 4.3. Reconstruction of Phylogenetic Tree

Phylogenetic relationships of IgH sequences inside the B-cell lineage were inferred by the DNA parsimony method implemented as the “dnapars” command of PHYLIP v3.697 software package [39] called from the Alakazam module (v1.2.0) of Immcantation framework. Relationship structure was visualized as a tree-shaped graph using the igraph (v1.3.4) R library (G. Csardi and T. Nepusz).

## Supplementary Materials

The following supporting information can be downloaded at https://www.mdpi.com/article/10.3390/ijms24010225/s1. The AIRRMINE repertoire data analysis software package including the ImmChainTracer pipeline can be accessed at https://github.com/blazsop/airrmine.
